# Case Management for Patients with Complex Multimorbidity: Development and Validation of a Coordinated Intervention between Primary and Hospital Care

**DOI:** 10.5334/ijic.2493

**Published:** 2017-06-20

**Authors:** Salvador Tortajada, María Soledad Giménez-Campos, Julia Villar-López, Raquel Faubel-Cava, Lucas Donat-Castelló, Bernardo Valdivieso-Martínez, Elisa Soriano-Melchor, Amparo Bahamontes-Mulió, Juan M. García-Gómez

**Affiliations:** 1 Instituto de Investigación Sanitaria La Fe, ES; 2 Hospital Universitario y Politécnico La Fe, ES; 3 Universitat de València, ES; 4 Universidad Politécnica de Valencia, ES

**Keywords:** integrated care, case management, complex multimorbidity, chronic patient, hospital at home

## Abstract

In the past few years, healthcare systems have been facing a growing demand related to the high prevalence of chronic diseases. Case management programs have emerged as an integrated care approach for the management of chronic disease. Nevertheless, there is little scientific evidence on the impact of using a case management program for patients with complex multimorbidity regarding hospital resource utilisation.

We evaluated an integrated case management intervention set up by community-based care at outpatient clinics with nurse case managers from a telemedicine unit. The hypothesis to be tested was whether improved continuity of care resulting from the integration of community-based and hospital services reduced the use of hospital resources amongst patients with complex multimorbidity.

A retrospective cohort study was performed using a sample of 714 adult patients admitted to the program between January 2012 and January 2015. We found a significant decrease in the number of emergency room visits, unplanned hospitalizations, and length of stay, and an expected increase in the home care hospital-based episodes. These results support the hypothesis that case management interventions can reduce the use of unplanned hospital admissions when applied to patients with complex multimorbidity.

## Introduction

In recent years, healthcare systems have been facing a growing demand related to the high prevalence of chronic diseases and long-term conditions. International strategies advocates for new ways to provide high-complexity and patient-centred services in order to meet this growing demand [1,2]. A radical transformation of the care process is required to promote proactive care focused on health and stability rather than reactive attention and treatment. International studies suggest this approach can be supported with the implementation of innovative chronic care models [3,4], based on personalized treatments, sustainable health services, closed-loop relationships, better quality of services, and evidence-based medicine [5,6].

In particular, case management (CM) programs have emerged as an approach to the management of chronic disease focused on improving individuals’ health and serving social needs [7,8]. Nevertheless, there is controversial scientific evidence available about the impact of using CM programs for chronic patients in terms of healthcare utilisation, clinical variables or health outcomes. The difficulties and barriers for the evaluation of these interventions reside in the variability of activities that are developed under the term “case management” and the heterogeneity of disease profiles on patients under study [9]. In addition, the majority of publications analyse CM interventions on single chronic conditions [10,11,12] or on an elderly population [13,14] while the predominant and increasing norm in the population is the confluence of various chronic conditions [15]. The latter condition is better known as complex multimorbidity.

Multimorbidity is a term that can be defined as the “co-occurrence of three or more chronic conditions affecting three or more different body systems within one person without defining an index chronic condition” [16]. People suffering from multiple chronic conditions suffer from poor quality of life, disability, psychological distress and an increased mortality risk [17,18]. In addition, the multiple needs associated to multimorbidity exceed the individual impact on the healthcare system. This strain on health status and the healthcare system involves a large number of resources, and therefore requires the development of strategies aimed at the organization of integrated care for people under this condition [19].

Recent reviews have focused on determining the impact of CM on the outcomes in patients with complex multimorbidity and health systems [20,21]. The evidence about the effectiveness of interventions orientated to these patients remains limited, and the results do not provide enough evidence on which interventions are related to better outcomes. This is especially controversial in cost savings, which is the focus of interest of this article. Nevertheless, the analysis suggested elements that could improve the effect of CM interventions and could be interesting to be implemented in clinical practice.

In the systematic review of Smith et al. [20] the most common organizational interventions were identified as CM and multidisciplinary teamwork using the taxonomy defined by the Cochrane Effective Practice and Organization of Care [22]. In addition, the most common patient-oriented interventions were educational or self-management support-type ones. Although the results related to the utilization of services were unclear, the conclusions underlined that there is emerging evidence and a need for further studies focusing on multimorbidity on community settings. Likewise, working with a multidisciplinary team or involving a social worker has a beneficial effect in patient satisfaction and improves the effectiveness of CM intervention [21].

Since 2010, an integrated care intervention has been developed in our institution for patients with complex multimorbidity. The intervention is based on a CM program where the primary health care team and nurse case managers are the first line of care responsible for the follow-up. The program under study includes patient-centred education activities. In addition, the integrated care intervention has the support of the Hospital at Home (HaH) unit, which is focused on avoiding hospital admissions when the management of exacerbations is possible in the community.

In that sense, the main objective of this study is to analyse the effect of an integrated CM program designed for patients with complex multimorbidity in terms of healthcare resources utilisation. We hypothesized that a scheduled follow-up program together with the HaH support on the management of patients with complex multimorbidity could reduce health resources utilisation including unplanned hospital admissions, length of stay (LoS), and visits to emergency room.

## Methods

### Design and setting

We conducted an observational, retrospective cohort study using matched observations before and after the CM intervention, also called pre-post intervention design or quasi-experimental design [23]. The units of observation were adult patients with complex multimorbidity admitted to the CM program between January 2012 and January 2015.

### Population and inclusion/exclusion criteria

The sample under study was a cohort of chronic patients (N = 714) with complex multimorbidity. Patients were identified either during the hospital discharge process or in the community health care setting. In both cases, physicians in charge assessed predefined criteria for inclusion in the CM intervention. These criteria included adult patients with the presence of three or more chronic diseases with complex degrees of severity, including at least one of the following: heart failure, chronic renal failure, chronic pulmonary disease, neurological disease with moderate or severe permanent cognitive disability, and diabetes mellitus with target organ disease.

We excluded patients from the study when they were facing an end of life situation, and specific clinical conditions that required a different follow-up intervention: inflammatory bowel disease, amyotrophic lateral sclerosis, cystic fibrosis, hepatitis C, and/or mental health disease. In addition, we excluded patients under treatment with non-invasive ventilation devices and particular cases in which the adequacy of the real follow-up was not that established by the chronic CM program.

### Description of the Case Management general intervention

#### Initiation into the CM program: comprehensive assessment and patient empowerment

Once an individual was identified as a patient with complex multimorbidity, the program adapted the common elements of CM [5,8]. The initial phase of the CM Model included a comprehensive assessment performed by a physician and a nurse. The multidisciplinary team reviewed the background and current clinical and psychosocial status of the patient. The assessment included information about the ability to perform activities of daily living (using the Barthel Index) and cognitive functioning (using the Short Portable Mental Status Questionnaire), medication prescribed, social history, and care support. This assessment allowed the identification of problems and the resources available in the patient environment, such as support by an informal caregiver.

The initiation into the CM program was developed along a standard of three consecutive days through home visits and face-to-face meetings for 60 minutes approximately. Usually, the assessment was performed during the first day. On the remaining days, the nurse initiated the educational and preventive interventions based on the identified needs and the health-disease process.

Educational interventions were orientated towards empowering the patient from the beginning of the CM program and were maintained during the entire care plan since promoting self-care could contribute to achieving successful outcomes [24]. The educational contents included a separate description of each disease process, common signs and symptoms of exacerbation, and how to report them to the healthcare provider. A personalized educational pathway was developed integrating notions from all the clinical condition of interest for the patients, including relationships between diseases. Information leaflets with recommendations and a traffic light system with alert ranges for the main diseases (heart failure, COPD and diabetes) were developed and shared with patients.

Education related to prescribed medication: dosage, route, duration of treatment, and adverse effects was also included. Finally, the patients were encouraged to follow nutritional and exercise habits appropriate for each condition [25,26,27,28].

#### Development of the Case Management Care Plan

The CM programmed attention began after the patient was enrolled into the program. The objectives for the care plan, aligned with the initial assessment, are the promotion of self-care empowerment, treatment compliance, and the identification of risk situations by regularly monitoring signs and symptoms [29,30]. Clinicians established stability indicators and regular diagnosis techniques related to chronic diseases in accordance with the available evidence-based guidelines [31,32].

The Primary Health Care Team (PHCT), formed by a general practitioner and a community nurse, together with the nurse case manager were responsible for the scheduled follow-up period and the reference for the enrolled patients. The scheduled care plan for the PHCT was based on face-to-face meetings through home visits or consultations depending on the functional status and the needs of the patients. Patients received one contact every two months by each member of the team alternately.

At the same time, case manager nurses from the Telemedicine Unit were responsible for the remote follow-up through structured phone calls carried out every 15 days, consecutively. The interviews focused mainly on functional and clinical outcomes, monitoring of signs and symptoms of exacerbation of each disease, and reinforcing health recommendations to patients.

When potential social risk was identified, a social worker was involved in the care plan. The appointments with the outpatient clinic were continued as expected and planned by each specialist.

The reasons for case closure were non-adherence to the CM program, moving to a residence outside the region, or death.

Case manager nurses were also responsible for coordinating patients along community and hospital resources, especially on the transition from hospital to home after discharge or when potential risk situations were identified, triggering contacts from the PHCT.

One of the goals when facing probable disease exacerbation was to reduce the risk of unplanned admission to hospital. When possible, the initial approach was given by medical and nursing staff from the PHCT. If the clinical condition required more intensive attention in the community setting, the patient was admitted via the HaH unit.

The admission to the HaH unit offered a strong care alternative as these schemes incorporate many elements of hospital care into the community setting. Multidisciplinary teams were able to visit patients on a daily basis and perform advanced diagnostic and therapeutic techniques such as non-invasive breathing monitoring, endovenous treatments, or paracentesis, among others [33]. During the clinical follow-up, community based care resources gave a response until patients recovered. When disease exacerbation could not be managed or reverted at home, patients were admitted to the hospital.

The signature strengths of the developed program are on the one hand, promoting proactive care through Primary Health scheduled interventions together with the support of the Telemedicine Unit and on the other, recognizing and anticipating the early symptoms of an exacerbation in order to provide advanced care developed by the HaH Unit and try to reduce avoidable hospital admissions or length of stay by community-dwelling chronic patients.

### Statistical analysis and modeling

The goal of our study was to find out if the CM intervention had a positive effect in reducing the number of emergency room visits, the number of admissions and/or the LoS, considered as the sum of the length of stay of all the admissions of a patient. The statistical analyses were carried out mainly for hospital use and secondly for HaH use.

We analyzed the effectiveness of the CM intervention by comparing the resource utilization of the same patients before and after being included in CM. Thus, we have two groups: a pre-intervention group, where we take into account the number of admissions and LoS of the patients one year *before* their inclusion in the CM intervention; and an intervention group, where we take into account the number of admissions and LoS of the patients until their discharge from CM regardless of its cause. The subjects under study are thus matched. Moreover, the patient in the pre-intervention group is observed in a time-window of 365 days, while the same patient in the post-intervention group is likely to be observed in a different time-window (greater or less than 365 days). We normalized the time-window of the intervention group to one year by using the rate of admissions and the rate of the length of stays.

We used the nonparametric two-tailed matched Wilcoxon’s signed rank test with continuity correction for testing the null hypothesis that there is no effect when including a patient in the CM intervention.

We also calculated the Relative Risk (RR) and their confidence interval at 95% [34]. The RR has been calculated taking into account the whole time-window of every patient under study before and after the intervention.

#### Ethical aspects

This research did not imply any risk or changes in the healthcare services to patients, and did not alter their regular intervention and treatment. Only authorized people obtained data from electronic health records. We maintained the privacy and security of patients’ personal information by encoding their identity with dissociated non-traceable codes. This research was in accordance with the International Guideline for Ethical Review of Epidemiological Studies [35] and the Biomedical Research Ethics Committee of our institution, which approved the study protocol.

## Results

The sample under study had a mean age of 78.5, where 69.3% of the patients were over 75 years old. The percentage of women was 50.7%. The average number of systems affected by the multimorbidities of the patients was 4.95 and the average number of organs affected was 8, and the most prevalent affected system was the cardiac system. Around 1 out of 5 patients died during the CM intervention. Table 1 describes the socio-demographic features and comorbidity profile.

**Table 1:** Description of the sample under study (N = 714).

Feature	Mean or Percentage

Age	78.5
% > 75 years old	69.3 %
% of women	50.7 %
#Systems affected	4.95
#Organ systems affected per patient	8
Cardiology	73.2 %
Cancer	24.6 %
Diabetes	47.5 %
Pulmonary	36.9 %
Community Assessment Risk Screen^1^	5.53
% CARS > 4	71.3 %
Mortality	21.6 %

^1^ The Community Assessment Risk Screen (CARS) is a tool for identifying community dwelling elderly patients at increased risk (CARS > 4) for hospitalizations or emergency room visits [36].

The information about the hospital resource utilization before and after CM intervention is shown in Table 2. It includes the minimum, maximum and mean number of each outcome normalized for one year. The original values of the hospital resource utilization after intervention are shown in Table 3.

**Table 2:** Hospital resource utilization normalized for one year.

Outcome	Pre-intervention			CM Intervention		

	Min	Mean	Max	Min	Mean	Max

Days of follow-up	–	365	–	–	365	–
Emergency room visits	0	2.54	17	0	1.87	16.92
Unplanned admissions	0	1.01	9	0	0.58	7.69
LoS due to unplanned admissions	0	10.21	248	0	5.31	103.82
HaH admissions	0	0.68	10	0	1.02	23.84
LoS at HaH	0	9.19	82	0	4.38	74.60

**Table 3:** Original values for hospital resource utilization after the CM intervention.

Outcome	CM Intervention		

	Min	Mean	Max

Days of follow-up	2	474.6	1102
Emergency room visits	0	2.43	22
Unplanned admissions	0	0.76	10
LoS due to unplanned admissions	0	6.91	135
HaH admissions	0	1.32	31
LoS at HaH	0	5.70	97

The results of the two-tailed matched Wilcoxon’s hypothesis test, with a significance level at α = 5%, showed statistically significant differences for the rate of unplanned admissions (median difference 0.23, CI 95% [0.08, 0.38]) and for the rate of emergency room visits (median difference 0.28 [0.10, 0.50]). However, the rate of admissions in HaH showed a significant increase (median difference –0.59 [–0.91, –0.36]). In the case of LoS, the results showed statistically significant differences for the rate of unplanned hospitalization LoS (median difference 2.75 [1.25, 4.27]).

**Figure 1:**
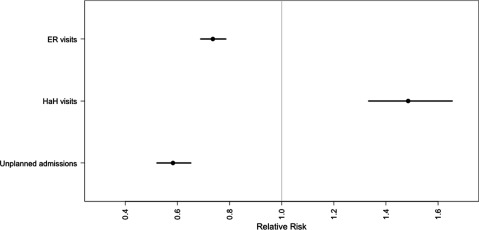
The relative risks and 95% confidence interval for unplanned hospital admission, HaH episodes, and visits to the Emergency room.

The RR reduction for unplanned admission is 58.4% (RR = 0.584 with 95% confidence interval [0.522, 0.652]), and the RR reduction for Emergency Room visits is 73.5% (RR = 0.735, with CI95% [0.688, 0.785]). However, the HaH admission RR increases by 50.2% (RR = 1.502, with CI95% [1.346, 1.675]). Figure 1 shows the results.

## Discussion

Nowadays, confronting the growing burden of chronic disease poses a major challenge for health care and social policies. A wide variety of strategies, orientated to dealing with this challenge, have been evaluated. However, the results are still insufficient to demonstrate significant benefits on hospital resources utilization [20,21].

The program under validation is based on a CM intervention focused on patients with complex multimorbidity which works on synergy between primary and hospital care resources. The intervention under study faces the problem of frequent hospital uses by high-risk patients in two main areas: the promotion of proactive interventions and the possibility of early and intensive care at home.

The one-year normalization allowed us to carry out a balanced comparison of the before-after group. Our results reveal that the CM program reduces the number of unplanned admissions and the visits to the emergency room. Furthermore, when the patient has to be admitted to the hospital, the mean number of LoS is reduced.

The variety of needs expressed by people with multimorbidity requires complex and multifaceted interventions. We consider that the simultaneous application of organizational and educational initiatives has been essential for reducing unexpected health care resources use [22].

The program promotes proactive attention through educational interventions initiated upon admission into the program and reinforced by the case manager nurses during the scheduled follow-up period. It empowers patients on self-care, which may benefit the early identification of risk situations [37,38]. The evidence however is still unclear about the effect of health self-management training on hospital resource utilization [20].

In addition, we believe that the scheduled contacts facilitate accessibility to medical attention. Also, the involvement of specific nursing roles [39,40,41] and social workers [21] together with the primary healthcare team could have had a positive impact on the results.

Consistent with [11,12], we believe that the follow-up through structured telephone intervention developed by case managers could also have had an impact on reducing the risk of hospital resource utilization.

Apart from the aforementioned proactive interventions, the next signature strength pending for discussion is the role of the HaH unit when faced with unplanned clinical or social needs. The diagnostic and therapeutic possibilities allow early attention to the exacerbations and acute needs of chronic disease at home, which could explain the decrease in hospital and emergency use in the population under study. The role of the HaH managing relevant health status changes shares similar aspects of the *virtual ward* concept described by Lewis et al. [42].

Besides the function of the HaH unit for avoiding conventional hospitalizations, we also consider important their role in reducing LoS once the patient is admitted to hospital. In this sense, our results are aligned with those obtained in recent publications [43,44]. In addition, the results show an increase in the number of HaH admissions once the patient is included in the CM program. This result may be attributable to the design of the CM intervention itself, so it may indicate an appropriate use of Hospital-based home care as an alternative to conventional hospitalization for patients with complex multimorbidity.

## Limitations and future work

Since the CM intervention depends on the context, the adoption of this type of intervention to a different health center may require a careful implementation according to the characteristics of chronic patients.

The lack of information on primary care is a main limitation of this study. Since we could not access the data from primary care, it was impossible for us to determine the impact of the intervention on primary care resource utilization.

The aim of the study was to analyze the relation between integrated care intervention and hospital resource utilization. However, it would be interesting to know the effect of specific aspects of this multifaceted intervention. But the analysis did not allow us to determine the effects of each component of the model applied separately.

Despite the significant results obtained, quasi-experimental designs require careful analysis to reduce the plausibility of alternative causal explanations other than the CM intervention. Alternative explanations include the principle of regression to the mean, and also maturation effects. However, since the study was focused on individuals with complex multimorbidity, the evolution of the condition is likely to aggravate or even become a patient in an end-of-life situation, which would likely require more health care resources than before.

The next step should be to carry out a cost-effectiveness analysis to compare if the intervention implies economic savings apart from the observed and tested hospital resources savings.

## Conclusions

Since the prevalence of chronic diseases is growing in our society, there is an increasing need to provide sustainable, optimized and personalized healthcare for this population segment. CM intervention implemented at our institution has produced a significant hospital resource savings on average, with a positive impact on chronic patients with complex multimorbidities regarding admissions and LoS. We suggest that continuous and close monitoring by the primary healthcare team and the nurse case manager, together with the participation of other professionals such as a social worker or a Hospital at Home unit, constitute a multidisciplinary team, which may be able to face the needs of patients with complex multimorbidity in the community, avoiding hospital contacts. We consider that policies and strategies should keep promoting integrated care interventions both for research and for the healthcare networks of patients and families.

## References

[1] Nolte E, McKee M, Ellen N, Martin M (2008). Caring for people with chronic conditions: an introduction. Caring for people with chronic conditions. A health system perspective.

[2] World Health Organization (2005). Preventing chronic diseases: a vital investment.

[3] Healy J-C (2004). Integration and Informatics and Communication Technologies (ICT) in the EU national health systems: status and trends. Swiss Medical Informatics.

[4] Committee on Quality of Health Care in America, Institute of Medicine (2001). Crossing the quality chasm.

[5] Case Management Society of America (2010). Standards of Practice for Case Management.

[6] Reid PP (2005). Building a Better Delivery System: A New Engineering/Health Care Partnership.

[7] Woodward J, Rice E (2015). Case Management. Nursing Clinics of North America.

[8] Ross S, Curry N, Goodwin N (2011). Case management. What it is and how can it best be implemented. The King’s fund.

[9] Kumar GS, Klein R (2013). Effectiveness of case management strategies in reducing emergency department visits in frequent user patient populations: a systematic review. The Journal of Emergency Medicine.

[10] Fan VS, Gaziano JM, Lew R (2012). A comprehensive care management program to prevent chronic obstructive pulmonary disease hospitalizations: a randomized, controlled trial. Annals of Internal Medicine.

[11] Ferrante D, Varini S, Macchia A (2010). Long-Term Results After a Telephone Intervention in Chronic Heart Failure: DIAL (Randomized Trial of Phone Intervention in Chronic Heart Failure) Follow-Up. Journal of the American College of Cardiology.

[12] Clark RA, Inglis SC, McAlister FA, Cleland JGF, Simon S (2007). Telemonitoring or structured telephone support programmes for patients with chronic heart failure: systematic review and meta-analysis. BMJ.

[13] You EC, Dunt D, Doyle C, Hsueh A (2012). Effects of case management in community aged care on client and carer outcomes: a systematic review of randomized trials and comparative observational studies. BMC Health Services Research.

[14] Huntley AL, Thomas R, Mann M, Huws D, Elwyn G, Paranjothy S, Purdy S (2013). Is case management effective in reducing the risk of unplanned hospital admissions for older people? A systematic review and meta-analysis. Family Practice.

[15] Tinetti ME, Fried TR, Boyd CM (2012). Designing Health Care for the Most Common Chronic Condition—Multimorbidity. JAMA.

[16] Harrison C, Britt H, Miller G, Henderson J (2014). Examining different measures of multimorbidity, using a large prospective cross-sectional study in Australia general practice. BMJ Open.

[17] Zulman DM, Pal Chee C, Wagner TH, Yoon J, Cohen DM, Holmes TH, Ritchie C, Asch SM (2015). Multimorbidity and healthcare utilisation among high-cost patients in the US Veterans Affairs Health Care System. BMJ Open.

[18] Vogeli C, Shields AE, Lee TA, Gibson TB, Marder WD, Weiss KB, Blumenthal D (2007). Multiple Chronic Conditions: Prevalence, Health Consequences, and Implications for Quality, Care Management, and Costs. Journal of General Internal Medicine.

[19] Goodwin N, Sonola L, Thiel V, Kodner DL (2013). Co-ordinated care for people with complex chronic conditions. Key lessons and markers for success. The King’s Fund.

[20] Smith SM, Wallace E, O’Dowd T, Fortin M (2016). Interventions for improving outcomes in patients with multimorbidity in primary care and community settings. Cochrane Database of Systematic Reviews.

[21] Stokes J, Panagioti M, Alam R, Checkland K, Cheraghi-Sohi S, Bower P (2015). Effectiveness of Case Management for ‘at risk’ patients in primary care: systematic review and meta-analysis. PLOS One.

[22] The Effective Practice and Organisation of Care (EPOC) Group (2015). The EPOC taxonomy of health systems interventions.

[23] Harris AD, Bradham DD, Baumgarten M, Zuckerman IH, Fink JC, Perencevich EN (2004). The use and interpretation of Quasi-experimental studies in infectious disease. Antimicrobial resistance.

[24] Boren SA, Wakefield BJ, Gunlock TL, Wakefield DS (2009). Heart failure self-management education: a systematic review of the evidence. International Journal of Evidence-based Healthcare.

[25] Powell LH, Calvin JE, Richardson D (2010). Self-management counseling in patients with heart failure: the heart failure adherence and retention randomized behavioral trial. JAMA.

[26] Azzolin K, Mussi CM, Ruschel KB, de Souza EN, de Fátima Lucena A, Rabelo-Silva ER (2013). Effectiveness of nursing interventions in heart failure patients in home care using NANDA-I, NIC, and NOC. Applied Nursing Research.

[27] Zwerink M, Brusse-Keizer M, van der Valk P (2014). Self management for patients with chronic obstructive pulmonary disease. Cochrane Database of Systematic Reviews.

[28] Health Quality Ontario (2013). In-home care for optimizing chronic disease management in the community: an evidence-based analysis. Ontario Health Technology Assessment Series.

[29] Freund T, Baldauf A, Muth C, Gensichen J, Szecsenyi J, Peters-Klimm F (2011). Practice-based home visit and telephone monitoring of chronic heart failure patients: rationale, design and practical application of monitoring lists in the HICMan trial (English version). Zeitschrift für Evidenz, Fortbildung und Qualität im Gesundheitswesen.

[30] Riegel B, Carlson B, Kopp Z, LePetri B, Glaser D, Unger A (2002). Effect of a standardized nurse case-management telephone intervention on resource use in patients with chronic heart failure. Archives of Internal Medicine.

[31] Yancy CW, Jessup M, Bozkurt B (2013). ACCF/AHA guideline for the management of heart failure: a report of the American College of Cardiology Foundation/American Heart Association Task Force on Practice Guidelines. Journal of the American College of Cardiology.

[32] American Diabetes Association (2014). Standards of Medical Care in Diabetes—2014. Diabetes Care.

[33] Corrado OJ (2001). Hospital-at-Home. Age and Ageing.

[34] Sistrom CL, Garvan CW (2004). Proportions, Odds and Risk. Radiology.

[35] Council for International Organizations of Medical Sciences (2009). International Ethical Guidelines for Epidemiological Studies.

[36] Shelton P, Sager MA, Schraeder C (2000). The community assessment risk screen (CARS): identifying elderly persons at risk for hospitalization or emergency department visit. American Journal of Managed Care.

[37] Effing T, Monninkhof EEM, van der Valk PPDLPM, Zielhuis GGA, Walters EH, van der Palen JJ, Zwerink M (2007). Self-management education for patients with chronic obstructive pulmonary disease. Cochrane Database Systematic Review.

[38] Aguado O, Morcillo C, Delàs J (2010). Long-term implications of a single home-based educational intervention in patients with heart failure. Heart & Lung: The Journal of Acute and Critical Care.

[39] Corser W, Dontje K (2011). Self-management perspectives of heavily comorbid primary care adults. Professional Case Management.

[40] Sutherland D, Hayter M (2009). Structured review: evaluating the effectiveness of nurse case managers in improving health outcomes in three major chronic diseases. Journal of Clinical Nursing.

[41] Schraeder C, Fraser CW, Clark I (2008). Evaluation of a primary care nurse case management intervention for chronically ill community dwelling older people. Journal of Nursing and Healthcare of Chronic Illness.

[42] Lewis G, Bardsley M, Vaithianathan R, Steventon A, Georghiou T, Billings J, Dixon J (2011). Do ‘virtual wards’ reduce rates of unplanned hospital admissions, and at what cost? A research protocol using propensity matched controls. International Journal of Integrated Care.

[43] Fox JP, Vashi AA, Ross JS, Gross CP (2014). Hospital-based, acute care after ambulatory surgery center discharge. Surgery.

[44] Vashi AA, Fox JP, Carr BG, D’Onofrio G, Pines JM, Ross JS, Gross CP (2013). Use of Hospital-Based Acute Care Among Patients Recently Discharged From the Hospital. JAMA.

